# Childhood Cancer Risk in Hispanic Enclaves in California

**DOI:** 10.1007/s10903-025-01675-0

**Published:** 2025-04-03

**Authors:** Darcy Van Deventer, Zuelma A. Contreras, Shiwen Li, Chisom Iwundu, Beate Ritz, Myles Cockburn, Julia E. Heck

**Affiliations:** 1 Department of Epidemiology, Fielding School of Public Health, University of California, Los Angeles, USA; 2 College of Health and Public Service, University of North Texas, Denton, TX USA; 3 Department of Neurology, Geffen School of Medicine, University of California, Los Angeles, CA USA; 4 Department of Population and Public Health Sciences, Keck School of Medicine, University of Southern California, Los Angeles, CA USA

**Keywords:** Acculturation, Childhood cancer epidemiology, Hispanic enclave, Retinoblastoma, Rhabdomyosarcoma, Neighborhood, Area-level

## Abstract

Residence in Hispanic enclaves may be a proxy measure of acculturation. Since acculturation among Hispanic women has been associated with unhealthy behaviors in pregnancy and adverse birth and child health outcomes, we assessed whether living in Hispanic enclaves during pregnancy affects childhood cancer risk among Hispanics. Cancer cases (*n* = 6,111) were identified from the California Cancer Registry between 1988 and 2013. Control children (*n* = 124,443) were randomly selected from California birth records. Data from the US decennial census (1990, 2000), and the American Community Survey (ACS) from 2007 to 2011 was used to create an index measure of Hispanic enclaves by census tract. In multivariable logistic regression models, we estimated the effects of living in Hispanic enclaves on cancer risk among young Hispanic children overall and by maternal nativity. We found positive associations between rhabdomyosarcoma in offspring and maternal residence in the least enclave-like tracts [OR = 1.62, 95% CI: (1.06, 2.46)]. For children of foreign-born Hispanic mothers, residence in the least enclave-like tracts was associated with lower retinoblastoma odds [OR = 0.59, 95% CI: (0.38, 0.91)]. In Los Angeles County, residing in the least enclave-like neighborhoods was positively associated with rhabdomyosarcoma and Wilms’ tumor odds [OR = 2.71, 95% CI: (1.27, 5.79), OR = 2.23, 95% CI: (1.26, 3.94), respectively]. Overall residence in Hispanic enclaves did not have a uniformly beneficial effect, rather living outside of these enclaves was associated with lower odds of certain childhood cancers. However, there was substantial variation in risk by maternal nativity status and county of residence.

**Supplementary Information:**

The online version contains supplementary material available at 10.1007/s10903-025-01675-0.

## Introduction

The Hispanic Epidemiologic Paradox suggests Hispanics may have better or comparable health outcomes to their white counterparts despite experiencing greater socioeconomic disadvantage [1]. This health advantage has been shown to extend to perinatal outcomes, particularly among Mexican-origin women [2]. Although much of the literature has focused on differences between Hispanic and white mothers, there is evidence that differences exist among Hispanic women such that worse perinatal outcomes are observed in US-born compared to foreign-born women. For instance, studies have found that US-born Hispanic mothers have higher rates of low birthweight and preterm birth compared with foreign-born Hispanic mothers [3–5]. The health differential by birthplace is hypothesized to be related to acculturation. Even though income, education, and access to health care services are higher in second-generation than immigrant mothers, US-born Hispanic women are also more likely to adopt negative health behaviors such as tobacco and alcohol use similar to their white peers, and experience higher levels of stress [2, 6].

One measure of acculturation is residence in Hispanic enclaves — neighborhoods that maintain native cultural practices and are culturally and ethnically distinct from surrounding areas [7]. Spatial assimilation theory states that when immigrants arrive in the US they tend to settle together, but as they achieve greater economic and social resources and acculturate, they leave their ethnic enclaves for more ethnically mixed neighborhoods. Hispanics who live in these enclaves are more socially integrated, have larger and more diverse social networks, and exhibit lower prevalence of negative health behaviors, such as smoking during pregnancy [8, 9].

Since many childhood cancers are related to exposures during pregnancy or early infancy, and living in an enclave has been associated with improved health resources and behaviors during pregnancy, it is plausible that living in an ethnic enclave may affect childhood cancer risk. While some risk factors for childhood cancers would not be expected to be related to lifestyle factors associated with acculturation (e.g., ionizing radiation), other established and suspected risk factors have been related to both acculturation and childhood cancer (maternal drug use, breastfeeding, parental smoking, and diet) [10–13].

To our knowledge, the only study on childhood risk in Hispanic enclaves found an increased risk of death among children with acute lymphoblastic leukemia (ALL) [14]. Compared to children of US-born Hispanic women, children of foreign-born Hispanic women were at reduced risk for most central nervous system (CNS) tumors, neuroblastoma, and Wilms’ tumor [13]. We hypothesize that for cancers in which maternal health behaviors seem to play an important role, particularly those mentioned above which have a lower incidence among foreign-born Hispanics, living outside of these enclaves will increase the risk of childhood cancers due to poorer health behaviors and less social support during pregnancy.

## Methods

This study includes children from the Air Pollution and Childhood Cancers (APCC) study [15]. We linked cancer cases identified from the California Cancer Registry (CCR) (1988–2013) to California birth records (1983–2011) for children diagnosed at 5 years of age or younger. The APCC study was restricted to young children because perinatal exposures are likely to be most relevant for cancers diagnosed in early childhood. In the parent study, 89% of cases were successfully matched to their birth certificate by first and last name, date of birth, and when available, social security number. Given the rates of residential mobility in early childhood, it is likely that the children we were unable to match were those who moved to California after birth [16]. Controls were children without a cancer diagnosis before 6 years of age. Controls were frequency-matched by year of birth to all childhood cancer cases during the study period (20:1 matching rate) and randomly selected from all California birth certificates. The present analysis is limited to children of Hispanic mothers and children born in California. Control children were excluded if they died of any cause prior to age 6 (*n* = 743), were missing sex (*n* = 3), or were likely non-viable births (gestational age < 20 weeks and/or birthweight < 500 g) (*n* = 90). The final sample included 6,111 cases and 124,443 controls.

Childhood cancer types were classified according to their respective International Classification of Childhood Cancer, 3rd edition (ICCC-3) or International Classification of Diseases for Oncology (ICD-O-3) codes [17] (See Table 2). We included specific cancer types with at least 65 cases.

Covariate information was obtained from California birth certificates. Addresses were geocoded using our open-source geocoder with manual correction of unmatched addresses [18]. Exact home addresses were recorded on electronic birth certificates from 1998. Prior to 1998, only ZIP codes were available, and we geocoded the ZIP code centroid for those children. Children born 1983–1995 were assigned to US decennial census 1990 tract data, children born 1996–2006 were assigned to US decennial census 2000 tract data, and children born 2007–2011 were assigned to American Community Survey (ACS) 2007–2011 tract data.

We conducted a principal component analysis for each census time point to recreate a neighborhood-level Hispanic enclave index that has been previously used in CCR studies [19] based on the following variables: percent of Hispanic residents foreign-born, recent immigrants, linguistically isolated households, Spanish language speaking households that are linguistically isolated, and all language speakers with limited English proficiency. The enclave measure was then categorized into quintiles based on the distribution of census tracts in California. Neighborhoods that score higher on this index are more characteristic of Hispanic enclaves, which will henceforth be referred to as more enclave-like neighborhoods.

We collapsed quintiles further into three categories to increase our sample size with the lowest three quintiles (0–60%) indicating the least enclave-like neighborhoods, quintile 4 indicating intermediate, and the highest quintile indicating the most enclave-like neighborhoods. For our neighborhood socioeconomic status (nSES) measure, we used aggregate-level data from the CCR to create an nSES index at the census tract level, based on the distribution in controls [20]. To harmonize data derived from distinct time periods, all census tract data was converted to 2010 census tract boundaries using the Longitudinal Tract Data Base (LTDB) [21], which provides public-use tools to create estimates within 2010 tract boundaries for any tract-level data that are available for prior years.

We assessed the associations between residence in Hispanic enclaves and childhood cancer risk using logistic regression accommodating for clustering at the census tract level. We adjusted for our matching variable, year of birth, as well as potential confounders including maternal age, maternal nativity (foreign-born vs. US-born), census-based neighborhood SES, paternal ethnicity, and paternal age [11, 13, 22]. We also assessed effect modification by maternal nativity and geographic region.

We also conducted a series of sensitivity analyses. Since exact home address was only collected starting 1998, we conducted sensitivity analyses to assess the effect of living in Hispanic enclaves on childhood cancer risk in this subgroup. Given that traffic-related air pollution is a potential confounder for Hispanic enclave and childhood cancer risk, we additionally adjusted for carbon monoxide (CO) levels at the home address for the child’s first year among those with CO estimates [23, 24]. We also examined how each component of our index correlates with childhood cancers to understand the specific factors influencing cancer risk. We categorized each variable similarly to the enclave index (lowest three quintiles = low, intermediate = 4th quintile, and 5th quintile = high).

All statistical analyses were performed using R version R-4.2.0 (R Foundation). This study was approved by the institutional review boards of the University of California Los Angeles and the California State Committee for the Protection of Human Subjects. As this was a noncontact study, patient consent was not required.

## Results

Hispanic mothers who lived in more enclave-like neighborhoods had fewer years of formal education, were slightly younger, and were more likely to be foreign-born as compared with mothers who lived in less enclave-like neighborhoods. These mothers were also more likely to have a public source of payment for their prenatal care, higher parity, and were less likely to smoke during pregnancy. The fathers of Hispanic children living in more enclave-like neighborhoods were more likely to have lower education, be Hispanic, and be slightly younger. Generally, more enclave-like neighborhoods were of disproportionately lower SES and predominantly located in Los Angeles County (Table 1).

**Table 1:** Distribution of individual and neighborhood level characteristics among children of Hispanic mothers by ethnic Enclave tertile, birth years 1983–2011

Hispanic Enclave Index			
	*N* (%)		
	1 (low)	2 (intermediate)	3 (high)
**Maternal education**,** y**^**a**^			
8 or less	6013 (16.3)	6824 (22.1)	15,169 (29.7)
9 to 11	8290 (22.5)	8558 (27.7)	16,085 (31.5)
12	11,323 (30.7)	9076 (29.3)	12,781 (25.0)
13 to 15	6922 (18.8)	4421 (14.3)	4980 (9.7)
16 or more	3760 (10.2)	1624 (5.3)	1451 (2.8)
Missing	556 (1.5)	436 (1.4)	655 (1.3)
**Maternal race**			
White	38,760 (96.8)	32,990 (97.7)	55,871 (98.5)
Black	210 (0.5)	131 (0.4)	115 (0.2)
Asian/Pacific Islander	875 (2.2)	544 (1.6)	683 (1.2)
Native American	188 (0.5)	78 (0.2)	76 (0.1)
Missing	17 (0.0)	10 (0.0)	6 (0.0)
**Maternal age**,** y**			
<20	5373 (13.4)	4756 (14.1)	8476 (14.9)
20–29	22,034 (55.0)	19,500 (57.7)	32,721 (57.7)
30–34	8181 (20.4)	6059 (18.0)	9995 (17.6)
35+	4458 (11.1)	3433 (10.2)	5548 (9.8)
Missing	4 (0.0)	5 (0.0)	11 (0.0)
**Source of payment for prenatal care** ^**a**^			
Private	15,667 (42.5)	10,173 (33.0)	11,798 (23.1)
Public (Medi-Cal) or self-pay	20,629 (56.0)	20,412 (66.0)	38,714 (75.7)
Missing	568 (1.5)	353 (1.1)	609 (1.2)
**Maternal Nativity**			
Foreign-born	19,411 (52.7)	19,492 (63.0)	38,048 (74.4)
US-born	17,438 (47.3)	11,435 (37.0)	13,060 (25.6)
Missing	15 (0.0)	12 (0.0)	13 (0.0)
**Parity**			
0	14,768 (37.0)	11,862 (35.1)	19,216 (33.9)
1	12,144 (30.3)	9914 (29.4)	16,475 (29.0)
2 or more	13,124 (32.8)	11,963 (35.4)	21,049 (37.1)
Missing	14 (0.0)	14 (0.0)	11 (0.0)
**Multiple births**			
Single	39,199 (97.9)	33,057 (97.9)	55,708 (98.2)
Multiple	851 (2.1)	696 (2.1)	1043 (1.8)
**Prenatal care initiation**			
First trimester	30,169 (75.3)	25,533 (75.7)	42,018 (74.0)
After first trimester or no care	9343 (23.3)	7681 (22.8)	13,802 (24.3)
Missing	538 (1.3)	539 (1.6)	931 (1.6)
**Smoking during pregnancy** ^**b**^			
Yes	105 (1.5)	49 (0.8)	45 (0.5)
No	6924 (97.0)	5793 (97.9)	8791 (96.9)
Missing	112 (1.6)	76 (1.3)	237 (2.6)
**Birthweight**,** g**			
Low birthweight (< 2500)	2102 (5.3)	1805 (5.4)	3024 (5.3)
Normal birthweight (2500-<4000)	33,862 (84.6)	28,486 (84.4)	48,096 (84.8)
High birthweight (4000+)	4058 (10.1)	3441 (10.2)	5585 (9.8)
Missing	28 (0.1)	21 (0.1)	46 (0.1)
**Paternal education**,** y**^**a**^			
8 or less	6300 (17.1)	6935 (22.4)	14,911 (29.2)
9–11	6800 (18.5)	6987 (22.6)	13,193 (25.8)
12	11,032 (29.9)	9027 (29.2)	12,271 (24.0)
13–15	5700 (15.5)	3507 (11.3)	3867 (7.6)
16 or more	3985 (10.8)	1607 (5.2)	1462 (2.9)
Missing	3047 (8.3)	2876 (9.3)	5417 (10.6)
**Paternal ethnicity**			
Hispanic	30,073 (75.1)	29,108 (86.2)	51,672 (91.1)
Non-Hispanic	8023 (20.0)	2887 (8.6)	1974 (3.5)
Missing	1954 (4.9)	1758 (5.2)	3105 (5.5)
**Paternal age**,** y**			
<20	1817 (4.9)	1622 (5.2)	2656 (5.2)
20–29	17,186 (46.6)	15,146 (49.0)	25,201 (49.3)
30–34	8064 (21.9)	6291 (20.3)	10,184 (19.9)
35+	7273 (19.7)	5475 (17.7)	8405 (16.4)
Missing	2524 (6.9)	2405 (7.8)	4675 (9.1)
**Neighborhood SES**			
1 (low)	3573 (8.9)	10,631 (31.5)	39,485 (69.6)
2	11,418 (28.5)	11,112 (32.9)	13,156 (23.2)
3	10,158 (25.4)	8756 (25.9)	3067 (5.4)
4	8858 (22.1)	2645 (7.8)	743 (1.3)
5 (high)	5552 (13.9)	591 (1.8)	125 (0.2)
Missing	491 (1.2)	18 (0.1)	175 (0.3)
**Regions in CA**			
Los Angeles County	6965 (17.4)	12,447 (36.9)	31,130 (54.9)
Central Valley	11,366 (28.4)	5683 (16.8)	5614 (9.9)
Other	21,719 (54.2)	15,623 (46.3)	20,007 (35.3)

^a^ collected 1989+

^b^ collected 2007+

We observed an increased risk of rhabdomyosarcoma in the children comparing Hispanic mothers living in the least to the most enclave-like neighborhoods. (See Table 2 and Supplemental Fig. 1). We observed a suggestive decreased risk of bilateral retinoblastoma comparing Hispanic mothers living in the least to the most enclave-like neighborhoods. When stratifying by maternal nativity, we observed a lower risk of retinoblastoma among foreign-born Hispanic mothers living in the least enclave-like neighborhoods compared to the most enclave-like neighborhoods (Supplemental Table 2). When restricted to LA county, the associations between living in the least enclave-like neighborhoods and rhabdomyosarcoma [OR = 2.71, 95% CI: (1.27, 5.79)] and Wilms’ tumor [OR = 2.23, 95% CI: (1.26, 3.94)] were stronger than the overall results (Supplemental Table 3). Additional results examining effect modification by maternal nativity and geographic region are shown in supplemental Tables 1–3.

**Table 2:** ORs for childhood cancer risk in low vs. high enclave-like neighborhoods

			Low vs. High		
	ICC-3 or ICD-O-3 code	N in High (Reference)	N in Low	Crude OR^a^	Adjusted OR^b^
Controls		54,114	38,184		
Total Cancer Cases		2637	1866	1.00 (0.93, 1.08)	0.96 (0.88, 1.05)
Acute lymphoblastic leukemia	011	904	639	1.01 (0.91, 1.13)	1.01 (0.87, 1.17)
Acute myeloid leukemia	012	159	96	0.85 (0.66, 1.09)	0.75 (0.54, 1.05)
Astrocytoma	032	140	105	1.07 (0.82, 1.39)	1.06 (0.74, 1.52)
Ependymoma	031	51	37	1.02 (0.66, 1.56)	0.85 (0.48, 1.48)
Medulloblastoma	9470	40	38	1.34 (0.86, 2.10)	1.66 (0.94, 2.94)
Germ cell	101–105	87	61	0.99 (0.72, 1.36)	0.87 (0.56, 1.36)
Yolk sac tumors	9071	40	26	0.92 (0.57, 1.48)	0.83 (0.47, 1.47)
Teratoma	9080	37	31	1.18 (0.74, 1.88)	0.88 (0.47, 1.65)
Hepatoblastoma	071	72	53	1.03 (0.73, 1.46)	0.90 (0.56, 1.43)
Non-Hodgkin lymphoma	022–023	94	50	0.76 (0.53, 1.10)	0.77 (0.47, 1.29)
Neuroblastoma	041	195	156	1.13 (0.91, 1.40)	0.80 (0.61, 1.06)
Retinoblastoma	050	147	96	0.92 (0.70, 1.21)	0.72 (0.51, 1.02)
Unilateral		98	72	1.04 (0.76, 1.42)	0.82 (0.55, 1.20)
Bilateral		47	23	0.68 (0.41, 1.15)	0.49 (0.23, 1.03)
Soft tissue sarcomas	091–095	124	105	1.20 (0.92, 1.57)	1.41 (1.01, 1.96)
Rhabdomyosarcoma	091	68	69	1.44 (1.02, 2.02)	1.62 (1.06, 2.46)
Wilms’ tumor	061	168	148	1.25 (1.01, 1.55)	1.22 (0.93, 1.60)

^a^ adjusted for year of birth

^b^ adjusted for year of birth, maternal age, maternal nativity, census-based SES, paternal ethnicity, paternal age

When assessing the effect of Hispanic enclaves on childhood cancer risk in children born to Hispanic mothers starting in 1998 (Supplemental Table 4), we found that estimates were similar to those that we observed in the entire sample, but with wider confidence intervals, with the exception of astrocytoma, which showed an increased risk [OR = 1.54, 95% CI: (1.03, 2.31)] in the least compared to the most enclave-like neighborhoods. In models additionally adjusted for traffic-related air pollution during the child’s first year, the same overall pattern for cancer risk was seen (data not shown).

In sensitivity analyses, we examined the individual factors that contributed to the index, and overall patterns were similar with a few notable exceptions. We observed a decreased risk in hepatoblastoma associated with low [OR = 0.63, 95% CI: (0.40, 0.99)] versus high percent of Hispanic individuals. The risk of rhabdomyosarcoma was associated with low percent of Spanish language speaking households that are linguistically isolated compared to high [OR = 1.72, 95% CI: (1.08, 2.73)]; while estimates for all other components had wide confidence intervals which crossed the null. Lastly, medulloblastoma was associated with low [OR = 3.30, 95% CI: (1.44, 7.57)] versus high percent recent immigrants. We did not find associations between any cancer types and percent of foreign-born, percent linguistically isolated households or percent of all language speakers with limited English proficiency (See Supplemental Tables 5 to 10).

## Discussion

In this population-based sample of children born to Hispanic mothers in California, we found mixed effects of Hispanic enclaves on childhood cancer risk. We found a decreased risk of retinoblastoma, particularly for children of foreign-born mothers, living in the least enclave-like neighborhoods. Hepatoblastoma risk was also reduced in the least enclave-like neighborhoods for US-born Hispanic mothers. In contrast, rhabdomyosarcoma risk was elevated in the least enclave-like neighborhoods.

Given our previous findings on childhood cancer risk among Hispanics by maternal birthplace, we expected that the risk of CNS tumors, neuroblastoma, and Wilms’ tumor would be highest in the least enclave-like neighborhoods [13]. Although we observed an increased risk of astrocytoma in low enclave-like neighborhoods in the 1998 + born subgroup and of medulloblastoma in neighborhoods with a low proportion of recent immigrants, we did not find a consistently increased risk of CNS tumors in low enclave-like neighborhoods.

Although the most enclave-like neighborhoods have been associated with a lower prevalence of pregnancy smoking, they may have a higher prevalence of other negative prenatal health behaviors (e.g., lack of important nutrients in the maternal diet, exposure to harmful environmental agents, or exposure to occupational hazards) [25, 26], which could explain the comparatively lower risk of certain cancers in the least enclave-like neighborhoods. These factors may impact risk through cleaner home and occupational environments and/or through access to health-promoting resources in neighborhoods.

In contrast, Hispanic enclaves also may contribute to greater environmental exposures. Studies have shown that air pollutants are associated with increased risk of certain childhood cancers [23, 27, 28] and that high enclave-like neighborhoods have higher traffic exposure [29]. However, when we controlled for traffic-related air pollution, the effect estimates we observed persisted, suggesting that traffic pollution did not account for the differences we saw by enclave status, though the possibility of residual confounding by other pollution sources remains.

Rhabdomyosarcoma was the only cancer which we consistently found elevated in low enclave-like neighborhoods. Studies examining perinatal factors in relation to rhabdomyosarcoma found a few potential risk factors that are also associated with acculturation, including birth control use, low socioeconomic status, paternal smoking, and parental marijuana and cocaine use [30, 31]. Additionally, breastfeeding has been found to reduce the risk of rhabdomyosarcoma [32]. Lower levels of acculturation have been associated with higher rates of breastfeeding and male smoking as well as reduced use of marijuana, cocaine, and birth control [13, 33–36]. Apart from paternal smoking, these findings support the idea that residing in a high enclave-like neighborhood is protective against childhood cancer due to the more favorable behaviors of Hispanics, but the associations between these lifestyle factors and rhabdomyosarcoma have not been well studied. We previously found an increased risk of rhabdomyosarcoma with late or no prenatal care, but after adjustment for this variable our enclave results were unchanged [37]. A potential explanation for the increased risk of rhabdomyosarcoma in these neighborhoods is the inverse association for rhabdomyosarcoma that has been found for atopic exposures (i.e., day care attendance, allergies, hives), suggesting that this cancer may share risk factors with acute lymphoblastic leukemia (ALL) since a protective advantage of these factors for ALL has been consistently found [32]. One study that examined immigrant density among Hispanics in relation to wheezing found a protective effect of higher immigrant density, especially for poor children and those with foreign-born parents and guardians [38]. In sensitivity analyses evaluating individual components of the enclave index, the point estimates remained elevated but decreased in magnitude and the confidence intervals widened across the null, except for the analysis for percent of Spanish language speaking households that are linguistically isolated. This may indicate that this index component is more relevant than others for rhabdomyosarcoma risk.

We do not know why, among foreign-born mothers, the risk of retinoblastoma is lower in low enclave-like neighborhoods. Variations by county may reflect small sample sizes. However, given that the Hispanic population in Los Angeles has one of the highest global rates of all childhood cancers (combined), further investigation into environmental factors that may be prevalent in these neighborhoods and unique characteristics of enclaves in LA county are warranted [39].

This study was limited by the lack of detailed covariate information on parental characteristics and exposures, thus, confounding by known and unknown risk factors is possible. Furthermore, we did not have information to determine whether differences exist among foreign-born Hispanics based on duration of residence in the US, as well as among US-born Hispanics by degree of acculturation.

There is also a potential for misclassification of exposure as residence was ascertained using zip codes prior to 1998. However, when we examined the effect of enclaves in the subgroup with full residential address (1998+), most estimates were consistent with our results for the entire sample. In addition, we relied on address at birth, which may also be a source of misclassification if mothers moved during pregnancy. A review found that 9–32% of women in the United States and abroad, in studies from the 1980–2000s, move residence during pregnancy, although most moves are local (median distance, < 10 km) [40]. Since our neighborhood-level information was based on US census data, which was not available on a yearly basis, we relied on the assumption that neighborhoods remained stable in the span of 5 to 10 years. A study investigating residential mobility among childhood cancer cases in California found moderate to strong agreement between residential address at birth and diagnosis but concluded that there is still potential for exposure misclassification in the first year after birth [41]. The definition of a neighborhood is also subjective and census boundaries may not accurately reflect the neighborhood of an individual, however census tracts are commonly used neighborhood boundaries and considered to be homogenous with respect to various sociodemographic characteristics [42]. Additionally, there is the potential for confounding by self-selection, whereby those with certain risk factors for childhood cancers may be more likely to choose to reside in these enclaves.

Despite these various limitations, this study was the first to assess the effect of Hispanic enclaves in relation to childhood cancer risk. The neighborhood environment may be a determinant of cancer risk and may contribute to disparities in cancer outcomes and allow for targeted studies and interventions. Additionally, the population-based design also allowed for a comprehensive assessment of childhood cancer types.

In conclusion, we found that residence in Hispanic enclaves did not have a uniformly beneficial effect on childhood cancer. We were not able to confirm a consistently increased risk of the childhood cancers we hypothesized to be most related to health behaviors (CNS tumors, neuroblastoma, and Wilms’ tumor) based on our previous findings. The increased risk of rhabdomyosarcoma among those living in low enclave-like neighborhoods may be partially explained by associations found between rhabdomyosarcoma and several beneficial lifestyle behaviors observed in the Hispanic Paradox, including an increased rate of breastfeeding and lower use of marijuana and cocaine. There was substantial variation in our results depending on the nativity status of the mother and the region of residence within California which is likely a reflection of the distinct distributions of risk factors in these populations—particularly aspects of acculturation that are not measured by residence. This suggests that Hispanic enclaves in themselves may not be a useful predictor of risk, rather further investigations into perinatal risk and preventive factors within Hispanic enclaves by maternal nativity are needed to better understand the patterns in cancer risk we observed.

## Electronic supplementary material

Below is the link to the electronic supplementary material.

Supplementary Material 1
