# Supplementary material for: Childhood Cancer Risk in Hispanic Enclaves in California
Source: J Immigr Minor Health. 2025 Apr 3;27(3):472–9. doi: 10.1007/s10903-025-01675-0 (PMC12037673; doi:10.1007/s10903-025-01675-0)
Supplement: Supplementary file 1 — Supplementary Material 1 [file 10903_2025_1675_MOESM1_ESM.docx]

| **Supplemental Fig. 1.** Association between Hispanic enclave and childhood cancer risks.  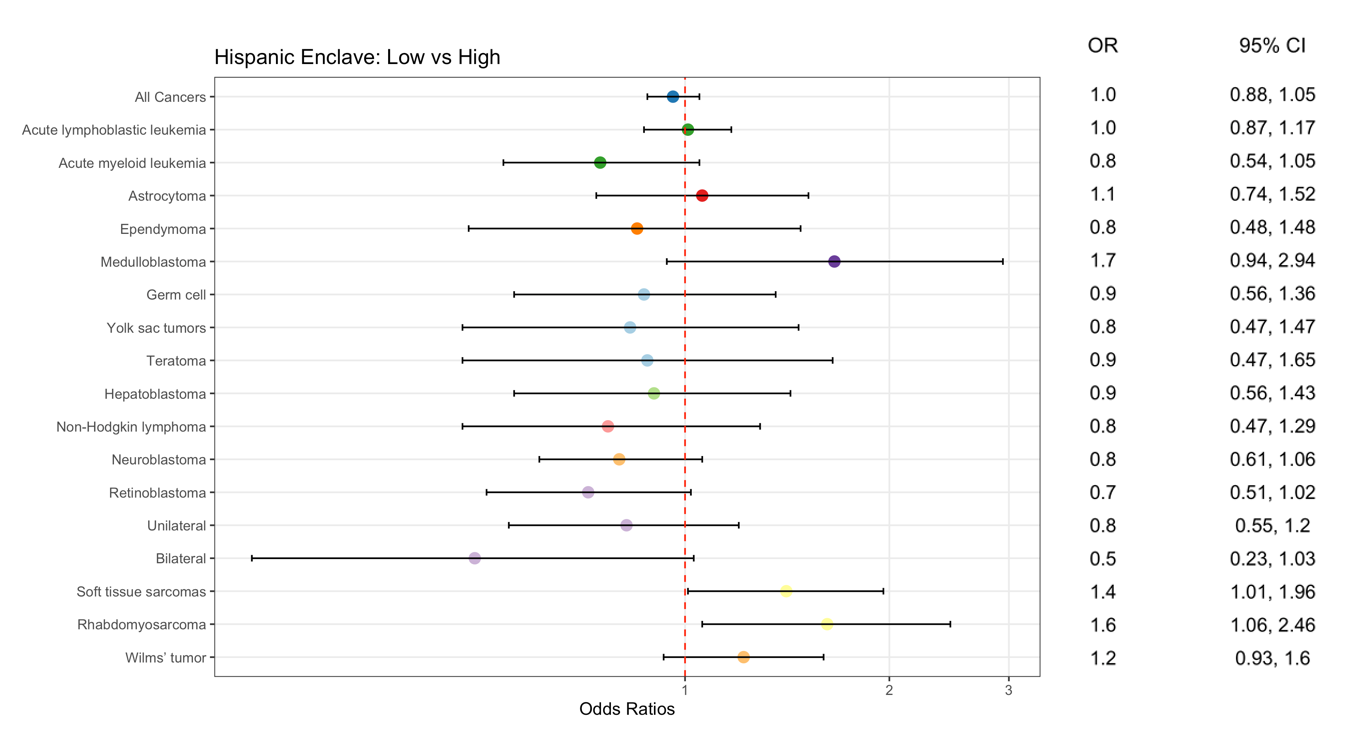  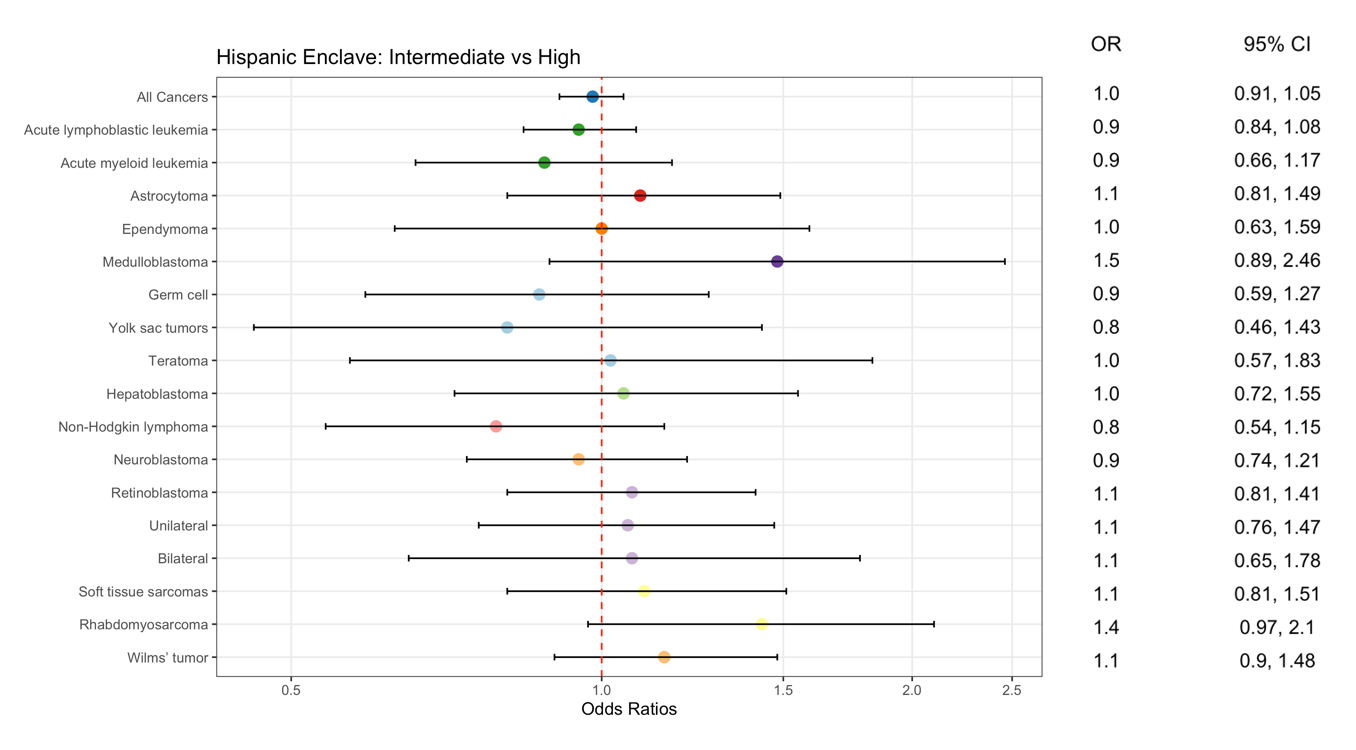 |
| --- |

| **Supplemental Table 1.** Adjusted ORs for childhood cancer risk among children of US-born Hispanic mothers by Hispanic enclave tertile | | | | |
| --- | --- | --- | --- | --- |
|  |  | **Low vs High** | | |
|  | N in High (Reference) | N in Low | Crude OR^a^ | Adjusted OR^b^ |
| Controls | 13719 | 18021 |  |  |
| Cases | 740 | 960 | 0.99 (0.89, 1.09) | 0.99 (0.87, 1.12) |
| Acute lymphoblastic leukemia | 234 | 333 | 1.09 (0.92, 1.29) | 1.13 (0.89, 1.42) |
| Acute myeloid leukemia | 47 | 45 | 0.73 (0.48, 1.10) | 0.67 (0.40, 1.11) |
| Astrocytoma | 44 | 63 | 1.09 (0.75, 1.60) | 1.32 (0.81, 2.15) |
| Ependymoma | 16 | 24 | 1.14 (0.61, 2.14) | 1.35 (0.63, 2.89) |
| Medulloblastoma | 14 | 22 | 1.20 (0.61, 2.34) | 1.62 (0.67, 3.88) |
| Germ cell | 21 | 27 | 0.98 (0.56, 1.69) | 0.78 (0.40, 1.54) |
| Yolk sac tumors | 5 | 12 | 1.82 (0.65, 5.12) | 1.48 (0.46, 4.72) |
| Teratoma | 12 | 14 | 0.89 (0.42, 1.89) | 0.66 (0.26, 1.64) |
| Hepatoblastoma | 26 | 21 | 0.61 (0.35, 1.08) | 0.44 (0.19, 1.01) |
| Non-Hodgkin lymphoma | 24 | 27 | 0.86 (0.48, 1.55) | 1.14 (0.52, 2.51) |
| Neuroblastoma | 65 | 76 | 0.89 (0.64, 1.24) | 0.72 (0.47, 1.10) |
| Retinoblastoma | 34 | 51 | 1.14 (0.73, 1.77) | 0.98 (0.55, 1.74) |
| Unilateral | 26 | 40 | 1.17 (0.71, 1.93) | 1.06 (0.58, 1.93) |
| Bilateral | 8 | 10 | 0.95 (0.37, 2.40) | 0.62 (0.14, 2.76) |
| Soft tissue sarcomas | 31 | 57 | 1.40 (0.91, 2.15) | 1.56 (0.94, 2.61) |
| Rhabdomyosarcoma | 18 | 36 | 1.52 (0.87, 2.67) | 1.51 (0.80, 2.86) |
| Wilms’ tumor | 49 | 75 | 1.17 (0.82, 1.66) | 1.08 (0.69, 1.67) |
| ^a^ adjusted for year of birth | | | | |
| ^b^ adjusted for year of birth, maternal age, maternal nativity, census-based nSES, paternal ethnicity, paternal age | | | | |

|  | | | | |
| --- | --- | --- | --- | --- |
| **Supplemental Table 2.** Adjusted ORs for childhood cancer risk among children of foreign-born Hispanic mothers by Hispanic enclave tertile | | | | |
|  |  | **Low vs High** | | |
|  | N in High (Reference) | N in Low | Crude OR^a^ | Adjusted OR^b^ |
| Controls | 40377 | 20146 |  |  |
| Cases | 1896 | 906 | 0.96 (0.87, 1.05) | 0.93 (0.83, 1.04) |
| Acute lymphoblastic leukemia | 670 | 306 | 0.92 (0.80, 1.06) | 0.92 (0.77, 1.10) |
| Acute myeloid leukemia | 112 | 51 | 0.91 (0.66, 1.26) | 0.83 (0.54, 1.28) |
| Astrocytoma | 96 | 42 | 0.88 (0.61, 1.27) | 0.82 (0.52, 1.32) |
| Ependymoma | 35 | 13 | 0.74 (0.39, 1.41) | 0.57 (0.24, 1.35) |
| Medulloblastoma | 26 | 16 | 1.23 (0.67, 2.26) | 1.50 (0.69, 3.29) |
| Germ cell | 66 | 34 | 1.03 (0.68, 1.55) | 0.91 (0.51, 1.61) |
| Yolk sac tumors | 35 | 14 | 0.80 (0.44, 1.45) | 0.66 (0.32, 1.38) |
| Teratoma | 25 | 17 | 1.36 (0.74, 2.49) | 1.04 (0.44, 2.45) |
| Hepatoblastoma | 46 | 32 | 1.39 (0.89, 2.15) | 1.32 (0.79, 2.22) |
| Non-Hodgkin lymphoma | 70 | 23 | 0.66 (0.42, 1.05) | 0.58 (0.32, 1.07) |
| Neuroblastoma | 130 | 80 | 1.23 (0.94, 1.62) | 0.89 (0.62, 1.28) |
| Retinoblastoma | 113 | 45 | 0.80 (0.55, 1.15) | 0.59 (0.38, 0.91) |
| Unilateral | 72 | 32 | 0.89 (0.58, 1.36) | 0.67 (0.41, 1.08) |
| Bilateral | 39 | 13 | 0.67 (0.35, 1.27) | 0.46 (0.20, 1.05) |
| Soft tissue sarcomas | 93 | 48 | 1.03 (0.70, 1.52) | 1.32 (0.81, 2.15) |
| Rhabdomyosarcoma | 50 | 33 | 1.32 (0.82, 2.14) | 1.66 (0.91, 3.03) |
| Wilms’ tumor | 118 | 73 | 1.24 (0.93, 1.67) | 1.32 (0.90, 1.94) |
| ^a^ adjusted for year of birth | | | | |
| ^b^ adjusted for year of birth, maternal age, maternal nativity, census-based nSES, paternal ethnicity, paternal age | | | | |

| **Supplemental Table 3.** Adjusted ORs for childhood cancer risk among Hispanic mothers living in LA by Hispanic enclave tertile | | | | |
| --- | --- | --- | --- | --- |
|  |  | **Low vs High** | | |
|  | N in High (Reference) | N in Low | Crude OR^a^ | Adjusted OR^b^ |
| Controls | 29627 | 6617 |  |  |
| Cases | 1503 | 348 | 1.04 (0.91, 1.18) | 1.02 (0.83, 1.25) |
| Acute lymphoblastic leukemia | 517 | 111 | 1.00 (0.82, 1.23) | 1.26 (0.89, 1.78) |
| Acute myeloid leukemia | 85 | 20 | 1.04 (0.64, 1.69) | 0.65 (0.30, 1.40) |
| Astrocytoma | 78 | 10 | 0.59 (0.29, 1.18) | 0.64 (0.21, 1.97) |
| Ependymoma | 27 | 7 | 1.14 (0.51, 2.53) | 0.98 (0.31, 3.14) |
| Medulloblastoma | 26 | < 5 | ---- | ---- |
| Germ cell | 49 | 8 | 0.72 (0.34, 1.53) | 0.45 (0.18, 1.10) |
| Yolk sac tumors | 19 | < 5 | ---- | ---- |
| Teratoma | 21 | 6 | 1.29 (0.52, 3.21) | 0.54 (0.15, 1.88) |
| Hepatoblastoma | 44 | 10 | 0.95 (0.48, 1.88) | 0.65 (0.22, 1.96) |
| Non-Hodgkin lymphoma | 51 | 9 | 0.83 (0.41, 1.69) | 0.67 (0.27, 1.64) |
| Neuroblastoma | 110 | 34 | 1.37 (0.94, 2.00) | 0.94 (0.49, 1.81) |
| Retinoblastoma | 85 | 14 | 0.71 (0.40, 1.28) | 0.57 (0.26, 1.23) |
| Unilateral | 54 | 12 | 0.97 (0.52, 1.80) | 0.88 (0.40, 1.94) |
| Bilateral | 30 | < 5 | ---- | ---- |
| Soft tissue sarcomas | 69 | 22 | 1.44 (0.85, 2.43) | 2.04 (1.02, 4.07) |
| Rhabdomyosarcoma | 39 | 16 | 1.85 (1.04, 3.29) | 2.71 (1.27, 5.79) |
| Wilms’ tumor | 96 | 34 | 1.63 (1.13, 2.36) | 2.23 (1.26, 3.94) |
| ^a^ adjusted for year of birth | | | | |
| ^b^ adjusted for year of birth, maternal age, maternal nativity, census-based nSES, paternal ethnicity, paternal age | | | | |

| **Supplemental Table 4.** Adjusted ORs for childhood cancer risk among births after 1998 by Hispanic enclave tertile | | | | |
| --- | --- | --- | --- | --- |
|  |  | **Low vs High** | | |
|  | N in High (Reference) | N in Low | Crude OR^a^ | Adjusted OR^b^ |
| Controls | 30974 | 21963 |  |  |
| Cases | 1543 | 1115 | 1.02 (0.94, 1.11) | 0.94 (0.84, 1.05) |
| Acute lymphoblastic leukemia | 460 | 339 | 1.08 (0.93, 1.25) | 1.01 (0.83, 1.22) |
| Acute myeloid leukemia | 98 | 60 | 0.85 (0.62, 1.18) | 0.79 (0.52, 1.19) |
| Astrocytoma | 70 | 73 | 1.51 (1.09, 2.09) | 1.54 (1.03, 2.31) |
| Ependymoma | 34 | 27 | 1.11 (0.67, 1.83) | 1.03 (0.54, 1.96) |
| Medulloblastoma | 28 | 22 | 1.13 (0.64, 1.99) | 1.15 (0.56, 2.39) |
| Germ cell | 54 | 30 | 0.77 (0.49, 1.20) | 0.68 (0.39, 1.20) |
| Yolk sac tumors | 26 | 11 | 0.59 (0.29, 1.19) | 0.50 (0.23, 1.09) |
| Teratoma | 23 | 18 | 1.09 (0.59, 2.02) | 0.87 (0.36, 2.11) |
| Hepatoblastoma | 53 | 33 | 0.85 (0.55, 1.32) | 0.70 (0.39, 1.27) |
| Non-Hodgkin lymphoma | 54 | 25 | 0.68 (0.42, 1.10) | 0.61 (0.32, 1.17) |
| Neuroblastoma | 127 | 94 | 1.03 (0.79, 1.35) | 0.81 (0.57, 1.15) |
| Retinoblastoma | 91 | 57 | 0.87 (0.63, 1.21) | 0.70 (0.45, 1.10) |
| Unilateral | 61 | 41 | 0.94 (0.64, 1.39) | 0.79 (0.48, 1.30) |
| Bilateral | 28 | 15 | 0.75 (0.40, 1.40) | 0.48 (0.19, 1.24) |
| Soft tissue sarcomas | 73 | 65 | 1.26 (0.90, 1.77) | 1.37 (0.90, 2.09) |
| Rhabdomyosarcoma | 39 | 45 | 1.64 (1.07, 2.52) | 1.65 (0.98, 2.78) |
| Wilms’ tumor | 93 | 90 | 1.37 (1.02, 1.83) | 1.08 (0.73, 1.59) |
| ^a^ adjusted for year of birth | | | | |
| ^b^ adjusted for year of birth, maternal age, maternal nativity, census-based nSES, paternal ethnicity, paternal age | | | | |

| **Supplemental Table 5.** Adjusted ORs for childhood cancer risk by percent of Hispanics tertile | | | | |
| --- | --- | --- | --- | --- |
|  |  | **Low vs High** | | |
|  | N in High (Reference) | N in Low | Crude OR^a^ | Adjusted OR^b^ |
| Controls | 54114 | 38184 |  |  |
| Cases | 2637 | 1866 | 0.99 (0.93, 1.06) | 0.97 (0.89, 1.05) |
| Acute lymphoblastic leukemia | 904 | 639 | 1.03 (0.92, 1.16) | 1.02 (0.89, 1.18) |
| Acute myeloid leukemia | 159 | 96 | 1.03 (0.78, 1.36) | 1.00 (0.72, 1.38) |
| Astrocytoma | 140 | 105 | 0.98 (0.74, 1.32) | 0.99 (0.69, 1.41) |
| Ependymoma | 51 | 37 | 1.43 (0.85, 2.43) | 1.41 (0.78, 2.57) |
| Medulloblastoma | 40 | 38 | 1.37 (0.83, 2.25) | 1.54 (0.88, 2.68) |
| Germ cell | 87 | 61 | 1.01 (0.71, 1.43) | 0.92 (0.58, 1.45) |
| Yolk sac tumors | 40 | 26 | 0.77 (0.46, 1.31) | 0.75 (0.38, 1.45) |
| Teratoma | 37 | 31 | 1.56 (0.85, 2.87) | 1.16 (0.55, 2.46) |
| Hepatoblastoma | 72 | 53 | 0.75 (0.52, 1.06) | 0.63 (0.40, 0.99) |
| Non-Hodgkin lymphoma | 94 | 50 | 0.75 (0.52, 1.09) | 0.69 (0.44, 1.08) |
| Neuroblastoma | 195 | 156 | 1.25 (1.00, 1.58) | 1.01 (0.76, 1.35) |
| Retinoblastoma | 147 | 96 | 0.92 (0.71, 1.20) | 0.80 (0.57, 1.13) |
| Unilateral | 98 | 72 | 0.86 (0.63, 1.18) | 0.68 (0.44, 1.04) |
| Bilateral | 47 | 23 | 1.14 (0.70, 1.85) | 1.12 (0.62, 2.02) |
| Soft tissue sarcomas | 124 | 105 | 1.08 (0.79, 1.48) | 1.16 (0.81, 1.67) |
| Rhabdomyosarcoma | 68 | 69 | 1.17 (0.78, 1.74) | 1.10 (0.69, 1.74) |
| Wilms’ tumor | 168 | 148 | 1.09 (0.85, 1.39) | 1.03 (0.77, 1.38) |
| ^a^ adjusted for year of birth | | | | |
| ^b^ adjusted for year of birth, maternal age, maternal nativity, census-based nSES, paternal ethnicity, paternal age | | | | |

| **Supplemental Table 6.** Adjusted ORs for childhood cancer risk by percent of foreign-born tertile | | | | |
| --- | --- | --- | --- | --- |
|  |  | **Low vs High** | | |
|  | N in High (Reference) | N in Low | Crude OR^a^ | Adjusted OR^b^ |
| Controls | 54114 | 38184 |  |  |
| Cases | 2637 | 1866 | 1.02 (0.95, 1.10) | 0.98 (0.91, 1.07) |
| Acute lymphoblastic leukemia | 904 | 639 | 1.00 (0.89, 1.11) | 0.98 (0.87, 1.10) |
| Acute myeloid leukemia | 159 | 96 | 0.92 (0.69, 1.22) | 0.90 (0.66, 1.23) |
| Astrocytoma | 140 | 105 | 1.09 (0.83, 1.45) | 1.05 (0.76, 1.46) |
| Ependymoma | 51 | 37 | 1.27 (0.77, 2.11) | 1.19 (0.68, 2.09) |
| Medulloblastoma | 40 | 38 | 1.11 (0.68, 1.81) | 1.18 (0.70, 2.00) |
| Germ cell | 87 | 61 | 0.99 (0.70, 1.39) | 0.94 (0.63, 1.39) |
| Yolk sac tumors | 40 | 26 | 0.92 (0.54, 1.58) | 0.86 (0.48, 1.52) |
| Teratoma | 37 | 31 | 1.25 (0.72, 2.17) | 1.09 (0.57, 2.09) |
| Hepatoblastoma | 72 | 53 | 0.97 (0.66, 1.41) | 0.86 (0.56, 1.32) |
| Non-Hodgkin lymphoma | 94 | 50 | 0.80 (0.55, 1.15) | 0.89 (0.59, 1.35) |
| Neuroblastoma | 195 | 156 | 1.25 (0.99, 1.58) | 0.94 (0.73, 1.23) |
| Retinoblastoma | 147 | 96 | 1.01 (0.78, 1.32) | 0.92 (0.68, 1.23) |
| Unilateral | 98 | 72 | 1.23 (0.88, 1.73) | 1.09 (0.75, 1.59) |
| Bilateral | 47 | 23 | 0.71 (0.45, 1.12) | 0.63 (0.38, 1.05) |
| Soft tissue sarcomas | 124 | 105 | 1.03 (0.76, 1.40) | 1.06 (0.76, 1.47) |
| Rhabdomyosarcoma | 68 | 69 | 1.28 (0.85, 1.94) | 1.22 (0.79, 1.87) |
| Wilms’ tumor | 168 | 148 | 1.08 (0.85, 1.37) | 0.98 (0.75, 1.28) |
| ^a^ adjusted for year of birth | | | | |
| ^b^ adjusted for year of birth, maternal age, maternal nativity, census-based nSES, paternal ethnicity, paternal age | | | | |

| **Supplemental Table 7.** Adjusted ORs for childhood cancer risk by percent linguistically isolated households tertile | | | | |
| --- | --- | --- | --- | --- |
|  |  | **Low vs High** | | |
|  | N in High (Reference) | N in Low | Crude OR^a^ | Adjusted OR^b^ |
| Controls | 54114 | 38184 |  |  |
| Cases | 2637 | 1866 | 1.03 (0.95, 1.11) | 0.98 (0.90, 1.06) |
| Acute lymphoblastic leukemia | 904 | 639 | 1.05 (0.94, 1.17) | 1.01 (0.89, 1.16) |
| Acute myeloid leukemia | 159 | 96 | 0.83 (0.64, 1.08) | 0.76 (0.55, 1.04) |
| Astrocytoma | 140 | 105 | 1.16 (0.87, 1.54) | 1.23 (0.86, 1.74) |
| Ependymoma | 51 | 37 | 1.43 (0.84, 2.42) | 1.54 (0.83, 2.85) |
| Medulloblastoma | 40 | 38 | 1.39 (0.82, 2.33) | 1.46 (0.80, 2.66) |
| Germ cell | 87 | 61 | 1.04 (0.73, 1.48) | 1.00 (0.64, 1.55) |
| Yolk sac tumors | 40 | 26 | 1.03 (0.59, 1.79) | 1.08 (0.57, 2.06) |
| Teratoma | 37 | 31 | 1.13 (0.67, 1.93) | 0.84 (0.43, 1.66) |
| Hepatoblastoma | 72 | 53 | 0.98 (0.67, 1.43) | 0.83 (0.52, 1.32) |
| Non-Hodgkin lymphoma | 94 | 50 | 0.78 (0.53, 1.14) | 0.80 (0.51, 1.27) |
| Neuroblastoma | 195 | 156 | 1.06 (0.85, 1.32) | 0.76 (0.58, 1.00) |
| Retinoblastoma | 147 | 96 | 1.15 (0.88, 1.52) | 1.04 (0.75, 1.43) |
| Unilateral | 98 | 72 | 1.23 (0.88, 1.73) | 1.06 (0.71, 1.58) |
| Bilateral | 47 | 23 | 1.07 (0.65, 1.77) | 0.99 (0.56, 1.76) |
| Soft tissue sarcomas | 124 | 105 | 1.04 (0.77, 1.39) | 1.10 (0.78, 1.54) |
| Rhabdomyosarcoma | 68 | 69 | 1.24 (0.84, 1.85) | 1.25 (0.80, 1.95) |
| Wilms’ tumor | 168 | 148 | 1.08 (0.85, 1.37) | 0.93 (0.70, 1.24) |
| ^a^ adjusted for year of birth | | | | |
| ^b^ adjusted for year of birth, maternal age, maternal nativity, census-based nSES, paternal ethnicity, paternal age | | | | |

| **Supplemental Table 8.** Adjusted ORs for childhood cancer risk by percent of Spanish language speaking households that are linguistically isolated tertile | | | | |
| --- | --- | --- | --- | --- |
|  |  | **Low vs High** | | |
|  | N in High (Reference) | N in Low | Crude OR^a^ | Adjusted OR^b^ |
| Controls | 54114 | 38184 |  |  |
| Cases | 2637 | 1866 | 1.09 (1.01, 1.17) | 1.02 (0.94, 1.11) |
| Acute lymphoblastic leukemia | 904 | 639 | 1.04 (0.93, 1.17) | 0.98 (0.86, 1.13) |
| Acute myeloid leukemia | 159 | 96 | 0.90 (0.69, 1.17) | 0.87 (0.64, 1.19) |
| Astrocytoma | 140 | 105 | 1.28 (0.95, 1.73) | 1.34 (0.96, 1.87) |
| Ependymoma | 51 | 37 | 1.17 (0.72, 1.90) | 1.05 (0.60, 1.84) |
| Medulloblastoma | 40 | 38 | 1.27 (0.78, 2.07) | 1.30 (0.73, 2.32) |
| Germ cell | 87 | 61 | 1.18 (0.81, 1.72) | 1.28 (0.84, 1.96) |
| Yolk sac tumors | 40 | 26 | 1.18 (0.66, 2.09) | 1.25 (0.69, 2.28) |
| Teratoma | 37 | 31 | 1.19 (0.70, 2.03) | 1.14 (0.61, 2.13) |
| Hepatoblastoma | 72 | 53 | 0.98 (0.66, 1.47) | 0.81 (0.51, 1.29) |
| Non-Hodgkin lymphoma | 94 | 50 | 0.86 (0.60, 1.24) | 0.88 (0.59, 1.31) |
| Neuroblastoma | 195 | 156 | 1.06 (0.85, 1.33) | 0.81 (0.63, 1.05) |
| Retinoblastoma | 147 | 96 | 1.17 (0.88, 1.55) | 1.05 (0.77, 1.44) |
| Unilateral | 98 | 72 | 1.26 (0.89, 1.79) | 1.07 (0.73, 1.58) |
| Bilateral | 47 | 23 | 1.07 (0.66, 1.74) | 1.01 (0.58, 1.76) |
| Soft tissue sarcomas | 124 | 105 | 1.34 (0.98, 1.82) | 1.39 (0.98, 1.95) |
| Rhabdomyosarcoma | 68 | 69 | 1.76 (1.15, 2.70) | 1.72 (1.08, 2.73) |
| Wilms’ tumor | 168 | 148 | 1.31 (1.00, 1.71) | 1.14 (0.85, 1.53) |
| ^a^ adjusted for year of birth | | | | |
| ^b^ adjusted for year of birth, maternal age, maternal nativity, census-based nSES, paternal ethnicity, paternal age | | | | |

| **Supplemental Table 9.** Adjusted ORs for childhood cancer risk by percent of all language speakers with limited English proficiency tertile | | | | |
| --- | --- | --- | --- | --- |
|  |  | **Low vs High** | | |
|  | N in High (Reference) | N in Low | Crude OR^a^ | Adjusted OR^b^ |
| Controls | 54114 | 38184 |  |  |
| Cases | 2637 | 1866 | 1.04 (0.97, 1.12) | 0.99 (0.91, 1.07) |
| Acute lymphoblastic leukemia | 904 | 639 | 1.03 (0.93, 1.16) | 1.00 (0.87, 1.13) |
| Acute myeloid leukemia | 159 | 96 | 0.89 (0.67, 1.18) | 0.81 (0.58, 1.13) |
| Astrocytoma | 140 | 105 | 1.13 (0.85, 1.51) | 1.09 (0.77, 1.55) |
| Ependymoma | 51 | 37 | 1.51 (0.88, 2.59) | 1.49 (0.81, 2.76) |
| Medulloblastoma | 40 | 38 | 1.12 (0.70, 1.81) | 1.22 (0.70, 2.11) |
| Germ cell | 87 | 61 | 0.96 (0.68, 1.36) | 0.85 (0.55, 1.32) |
| Yolk sac tumors | 40 | 26 | 0.91 (0.54, 1.54) | 0.81 (0.43, 1.51) |
| Teratoma | 37 | 31 | 1.19 (0.68, 2.09) | 0.95 (0.47, 1.90) |
| Hepatoblastoma | 72 | 53 | 1.04 (0.70, 1.54) | 0.90 (0.56, 1.43) |
| Non-Hodgkin lymphoma | 94 | 50 | 0.76 (0.52, 1.10) | 0.79 (0.51, 1.24) |
| Neuroblastoma | 195 | 156 | 1.18 (0.94, 1.49) | 0.88 (0.67, 1.17) |
| Retinoblastoma | 147 | 96 | 1.11 (0.85, 1.45) | 1.00 (0.72, 1.38) |
| Unilateral | 98 | 72 | 1.17 (0.84, 1.63) | 1.00 (0.67, 1.49) |
| Bilateral | 47 | 23 | 1.05 (0.64, 1.74) | 0.99 (0.56, 1.75) |
| Soft tissue sarcomas | 124 | 105 | 1.00 (0.75, 1.33) | 1.08 (0.77, 1.51) |
| Rhabdomyosarcoma | 68 | 69 | 1.24 (0.84, 1.84) | 1.26 (0.81, 1.97) |
| Wilms’ tumor | 168 | 148 | 1.11 (0.88, 1.40) | 0.97 (0.74, 1.29) |
| ^a^ adjusted for year of birth | | | | |
| ^b^ adjusted for year of birth, maternal age, maternal nativity, census-based nSES, paternal ethnicity, paternal age | | | | |

| **Supplemental Table 10.** Adjusted ORs for childhood cancer risk by percent recent immigrants tertile | | | | |
| --- | --- | --- | --- | --- |
|  |  | **Low vs High** | | |
|  | N in High (Reference) | N in Low | Crude OR^a^ | Adjusted OR^b^ |
| Controls | 54114 | 38184 |  |  |
| Cases | 2637 | 1866 | 1.03 (0.95, 1.12) | 0.98 (0.90, 1.08) |
| Acute lymphoblastic leukemia | 904 | 639 | 0.96 (0.85, 1.09) | 0.92 (0.80, 1.05) |
| Acute myeloid leukemia | 159 | 96 | 0.84 (0.62, 1.15) | 0.84 (0.61, 1.17) |
| Astrocytoma | 140 | 105 | 1.02 (0.75, 1.38) | 0.95 (0.66, 1.36) |
| Ependymoma | 51 | 37 | 1.03 (0.58, 1.80) | 1.02 (0.54, 1.92) |
| Medulloblastoma | 40 | 38 | 2.31 (1.17, 4.55) | 3.30 (1.44, 7.57) |
| Germ cell | 87 | 61 | 0.85 (0.58, 1.24) | 0.73 (0.47, 1.14) |
| Yolk sac tumors | 40 | 26 | 0.70 (0.40, 1.23) | 0.59 (0.32, 1.11) |
| Teratoma | 37 | 31 | 1.20 (0.66, 2.18) | 0.92 (0.45, 1.89) |
| Hepatoblastoma | 72 | 53 | 1.25 (0.77, 2.04) | 1.14 (0.66, 1.96) |
| Non-Hodgkin lymphoma | 94 | 50 | 0.85 (0.58, 1.23) | 0.86 (0.56, 1.31) |
| Neuroblastoma | 195 | 156 | 1.13 (0.86, 1.50) | 0.89 (0.65, 1.21) |
| Retinoblastoma | 147 | 96 | 1.10 (0.78, 1.53) | 0.93 (0.65, 1.34) |
| Unilateral | 98 | 72 | 1.44 (0.94, 2.21) | 1.21 (0.77, 1.90) |
| Bilateral | 47 | 23 | 0.69 (0.41, 1.18) | 0.61 (0.34, 1.11) |
| Soft tissue sarcomas | 124 | 105 | 1.24 (0.89, 1.72) | 1.31 (0.91, 1.89) |
| Rhabdomyosarcoma | 68 | 69 | 1.48 (0.96, 2.27) | 1.47 (0.91, 2.37) |
| Wilms’ tumor | 168 | 148 | 1.16 (0.88, 1.52) | 1.08 (0.80, 1.46) |
| ^a^ adjusted for year of birth | | | | |
| ^b^ adjusted for year of birth, maternal age, maternal nativity, census-based nSES, paternal ethnicity, paternal age | | | | |
